# Ocular Toxicity of Belantamab Mafodotin, an Oncological Perspective of Management in Relapsed and Refractory Multiple Myeloma

**DOI:** 10.3389/fonc.2021.678634

**Published:** 2021-05-11

**Authors:** Ahsan Wahab, Abdul Rafae, Kamran Mushtaq, Adeel Masood, Hamid Ehsan, Maria Khakwani, Aqsa Khan

**Affiliations:** ^1^ Internal Medicine/Hospital Medicine Department, University of Alabama at Birmingham, Montgomery, AL, United States; ^2^ Internal Medicine Residency Program, McLaren Regional Medical Center, Flint, MI, United States; ^3^ Internal Medicine/Hospital Medicine Department, Northeast Internal Medicine Associates, LaGrange, IN, United States; ^4^ Hospital Medicine, TidalHealth Peninsula Regional, Salisbury, MD, United States; ^5^ Biomedical Sciences/Biohazardous Threat Agents & Emerging Infectious Diseases Department, Georgetown University, Washington, DC, United States; ^6^ Department of Medicine, Lahore Medical and Dental College, Lahore, Pakistan; ^7^ Department of Medicine, Fatima Jinnah Medical University, Lahore, Pakistan

**Keywords:** belantamab mafodotin, B-cell maturation antigen, ocular toxicity, multiple myeloma, management

## Abstract

Belantamab mafodotin (belamaf), an antibody-drug conjugate approved for the treatment of relapsed and refractory multiple myeloma (RRMM), is an anti B-cell maturation antigen (BCMA) agent. DREAMM-1, a first in-human trial of belamaf, reported several ocular toxicities requiring dose adjustments, dose delays and treatment discontinuations. In DREAMM-1, 53% of patients in part-1 and 63% of patients in part-2 had ocular toxicity. Similarly, 73% of patients in DREAMM-2 had keratopathy (71% in 2.5 mg/kg *versus* 75% in 3.4 mg/kg) with the most common symptoms being blurred vision and dry eyes. Ocular toxicity of belamaf is attributed to microtubule-disrupting monomethylauristatin-F (MMAF), a cytotoxic payload of the drug that causes an off-target damage to the corneal epithelial cells. Ocular adverse events (AEs) of belamaf are more frequent at higher doses compared with lower doses. Higher belamaf dose, history of dry eyes and soluble BCMA are associated with increased risk of corneal toxicity. Absence of ocular symptoms does not exclude the possibility of belamaf-induced ocular toxicity, so patients need slit lamp and Snellen visual acuity testing to detect microcytic-like epithelial changes and visual decline. Corticosteroid eyes drops for 4-7 days prior to belamaf dose do not prevent ocular AEs and may cause steroid-related AEs instead. Keratopathy and Visual Acuity scale (*KVA*) is recommended to document the severity of belamaf-induced ocular toxicity and make treatment adjustments. Management of toxicity includes dosage modifications, treatment interruption or discontinuations and preservative-free artificial tears along with close ophthalmology and hematology-oncology follow-ups.

## Introduction

Belantamab mafodotin (belamaf, GSK2857916) is an antibody-drug conjugate (ADC) approved by the Food and Drug Administration (FDA) in August 2020 for the treatment of relapsed and refractory multiple myeloma (RRMM) (1). It counteracts B-cell maturation-antigen (BCMA) activity in multiple myeloma (MM). BCMA glycoprotein is naturally expressed as a cell surface transmembrane receptor of B-cells along with its ligands including proliferation-inducing ligand (APRIL) or B-cell activating factor (BAFF). BCMA regulates the differentiation of B-cells into both benign and malignant plasma cells (PCs) and is required for their longevity and survival (2, 3). Malignant PCs have a higher BCMA expression compared with non-malignant PCs. Its expression is also restricted on other organs making it an attractive target for MM therapeutics. The evidence from *in-vitro* and *in-vivo* studies indicates that overly expressed BCMA augments the proliferation and survival of malignant PCs and generates a bone marrow microenvironment that is conducive to myeloma cells proliferation (4).

BMA117159/DREAMM-1 (DRiving Excellence in Approaches to Multiple Myeloma-1) was the first in-human belamaf trial, the results of which were published in November 2018 (5). Investigators split the trial into two parts, i.e., dose-escalation and dose-expansion. There were no dose-limiting toxicities reported in the dose-escalation part, however corneal events occurred in 53% of patients. In the dose-expansion part, corneal events occurred even more frequently at 63% (5). These adverse events (AEs) were diverse including blurred vision, dry eyes, photophobia/eye pain, abnormal visual activity, and keratitis. The majority of the events were mild but caused frequent dose adjustments in the dose-expansion part. Therefore, although the results of DREAMM-1 were promising (≥ PR: 60.0%; 95% CI, 42.1–76.1, VGPR: 40%, CR: 9% and sCR: 6%, median progression-free survival: 12 months; 95% CI, 3.1-not-reached), ocular toxicity was recognized as an emerging concern, especially with larger belamaf doses (6). That is why the DREAMM-2 evaluated both the 3.4 mg/kg dose (recommended phase-2 dose (RP2D) in DREAMM-1) and 2.5 mg/kg dose (lower than RP2D) of belamaf for comparison (7). The patients in DREAMM-2 underwent systematic ocular history collection, National Eye Institute Visual Function questionnaire-25 (NEI-VFQ-25) and ophthalmic examinations. As a result of feedback from the FDA, ocular events were thoroughly evaluated in DREAMM-2 and the goal was successful development of ocular toxicity scale to guide toxicity mitigation and management strategies. Future DREAMM sequels will evaluate the role of belamaf in RRMM. This brief review focuses on belamaf-induced ocular toxicity, its mechanism of action, presentation, patient’s perspectives, preventive, and management strategies.

### Mechanism of Ocular Toxicity

Microtubule-disrupting monomethylauristatin-F (MMAF) is the cytotoxic component of belamaf that is linked to a monoclonal antibody *via* protease-resistant maleimidocaproyl (mc) linker. MMAF is proposed as an attributable cause of ocular toxicity along with other ADCs that used the MMAF (8, 9). ADCs may cause ocular toxicity *via* on-target or off-target processes (10). Belamaf has been previously detected in animal tears, circumstantially pointing toward its off-target damage site as the cornea lacks BCMA (11). Factors implicated to promote off-target ocular toxicity by ADCs may include linker instability or premature cleavage in extracellular environments, linker-cytotoxin intracellular metabolism, and Fc-receptor mediated cellular uptakes (10, 12). These mechanistic aspects of ocular toxicity caused by MMAF-containing ADCs may contribute to the ocular toxicity effects of belamaf. Though belamaf has a non-cleavable linker of maleimidocarproyl (mc) that provides resistance against degradation in extracellular environment, the combination of mc-MMAF in various ADCs such as SGN-75 and SGN-CD19A has known evidence of ocular toxicity (9, 13). One example of linker-cytotoxin metabolism in mc-MMAF is the intracellular liberation of ionized cytotoxic metabolite that is not capable of diffusing across the cell membrane and hence may promote localized cytotoxicity due to intracellular entrapment (10). Belamaf induces apoptosis of myeloma cells but may cause a concomitant off-target apoptosis of corneal epithelial cells due to microtubulin inhibition caused by MMAF. These corneal changes mirror as microcyst-like corneal epithelial changes (MECs) or keratopathy on slit lamp (11). MECs may occur following the first dose of belamaf but usually occur more frequently after subsequent exposures. MECs require medium to high magnification power when examined *via* slit lamp. On *in-vivo* confocal microscopy these changes may appear as hyper-reflective opacities (11). When belamaf encroaches the cornea *via* limbus vasculature or tears, it undergoes the process of solute internalization or cellular uptake *via* macropinocytosis (i.e., the process of pericellular belamaf eating without receptor-ligand interaction on cell surface) into corneal basal epithelial layer. Once belamaf is internalized into corneal cells it inhibits their proliferation eventually leading to their apoptosis. These corneal cells with swallowed-up belamaf travel away from the basal layer of cornea and approach its anterior or central parts and reflect their apoptosis with subsequent extrusion of dead cells (11). The migration of belamaf-carrying cells in the line of visual axis interferes with visual activity and causes ocular symptoms depending on their corneal location. Belamaf-containing cells and MECs are initially found at the periphery of cornea but eventually migrate in a centripetal fashion and then vanish due to extrusion (14). Theoretically, the inhibition of belamaf macropinocytosis might reduce occurrence of ocular toxicity but the practical role of such inhibition is limited. In animal and laboratory studies, the inhibition of macropinocytosis occurs *via* inhibition of membrane ruffle formation due to certain pharmacological agents such as imipramine (15). However, the clinical significance of such drugs remains unexplored in *in-vivo* and clinical trials. The mechanism of ocular toxicity requires further elucidation.

### Presentation of Ocular Toxicity

In part-1 of DREAMM-1 trial where belamaf dose ranged between 0.03 mg/kg to 4.60 mg/kg, the ocular toxicity occurred more frequently at larger doses than at smaller doses (5). Ocular toxicity occurred in 63% of patients in part-2 who received belamaf dose of 3.40 mg/kg. Blurred vision was reported in 29% (n=11) of patients in part-1 *versus* 46% (n=16) in part-2. Dry eyes were reported in 24% (n=9) of patients in part-1 *versus* 34% (n=12) in part-2 of DREAMM-1. In DREAMM-2, keratopathy was the most common ocular toxicity (73%, n=141) irrespective of its grades (71% in 2.5 mg/kg *versus* 75% in 3.4 mg/kg) and the most common complaints were also blurred vision (22% for 2.5 mg/kg *versus* 30% for 3.4 mg/kg) and dry eyes (14% for 2.5 mg/kg *versus* 23% for 3.4 mg/kg) (7). The patients with a prior history of dry eyes were more prone to develop corneal changes as shown in DREAMM-2.

When corneal changes occur in the form of keratopathy, the majority of patients are symptomatic. The absence of corneal symptoms, however, does not rule out the existence of keratopathy as indicated by the slit lamp and visual acuity testing data of DREAMM-2 participants. In the dosing cohort of 2.5 mg/kg of DREAMM-2, 72% of patients had MECs and 54% had vision changes. Contrarily, only 25% of those patients reported blurred vision and 15% reported dry eyes. Similarly, in the 3.4 mg/kg cohort of belamaf, 77% of patients had MEC, but 33% of those reported blurred vision and 25% reported dry eyes (7). This means that ocular toxicity requires active surveillance irrespective of symptoms. Even patients with grade (G)-3 or 4 keratopathies, i.e., severe superficial keratopathy and corneal ulcers may be asymptomatic. Such patients may continue to receive belamaf with ongoing toxicity unless screened *via* slit lamp (11). Keratopathy may occur somewhere between 9 days to 9 months after receiving belamaf with a median of 36 days. These events resolved in 36% of patients at median of 71 days (range, 57-99) in the dosing cohort of 2.5 mg/kg and in 28% of patients at median of 96 days (range, 70-127) in the dosing cohort of 3.4 mg/kg (7).

The exposure-safety analyses of DREAMM-2 evaluated the likelihood of G-2/G-3 corneal AEs and their relationship with belamaf concentration (16). Higher belamaf Ctau (the predicted concentration on day 21 at the end of first cycle) was associated with a lower threshold of developing G-2/G-3 corneal AEs in addition to an earlier onset of these events. A history of dry eyes and lower baseline serum concentration of soluble BCMA (sBCMA) were associated with an increased risk of G-2/G-3 corneal AEs. A history of dry eyes and baseline keratopathy prior to belamaf use were associated with a higher risk of any grade of blurred vision. Baseline keratopathy was also associated with an earlier onset of blurred vision along with an increased probability of ≥2 G-2 blurred vision (16).

The Common Terminology Criteria for Adverse Events (CTCAE v5), usually employed to grade AEs based on patients’ symptoms and interference with daily functioning, may under-estimate the severity of ocular toxicity (17). Due to the limitations of CTCAE, *Keratopathy and Visual Acuity (KVA)* scale devised in DREAMM-2 (11) outlines the objective findings of slit-lamp examination and best corrected visual acuity (BCVA) regardless of symptoms. Eighteen percent (n=17) of DREAMM-2 patients (n=97) in dosing cohort of 2.5 mg/kg experienced a BCVA decline to 20/50 or worse. The transient BCVA decline was relatively common with definite worsening in a considerably smaller proportion, i.e., 82% (n=14) *versus* 18% (n=3) (11). Two patients had BCVA decline to 20/200, which is considered to be legally blind in the United States (11, 18). It is important to consider the residual ocular sequelae of previous lines of therapies among patients who receive belamaf. About 67% of patients (n=49) in DREAMM-1 and 83% of patients (n=163) in DREAMM-2 had received at least 5 lines of prior therapies (5, 7). One hundred percent patients in DREAMM-1 and 98% of patients in DREAMM-2 had received bortezomib (5, 7). Bortezomib has previously been reported to cause eye disorders such as chalazion, blepharitis, and conjunctivitis (19).

The ocular health data of DREAMM-2 shows a huge burden of ocular problems in RRMM patients prior to belamaf use perhaps due to previously used steroids and bortezomib (20). These ocular abnormalities included 60% prevalence of cataract and 43% prevalence of corneal epithelial abnormalities followed by 20% blepharitis and 6% prevalence of glaucoma. Such a poor baseline ocular status of RRMM population might be partly responsible for their predisposition to higher rates of ocular toxicity seen in DREAMM-2 in addition to belamaf. Notably, the increased number of blepharitis and dry eyes in these patients may be associated with prior bortezomib use (20). In an ongoing DREAMM-6 study (NCT03544281), belamaf is being used in combination with lenalidomide-dexamethasone (Arm-A) or bortezomib-dexamethasone (Arm-B). As of March 30, 2020, 18 patients had received treatment in parts-1 & 2 of Arm-B, i.e., belamaf-bortezomib-dexamethasone. The AEs related to cornea/eye were responsible for dose interruptions or delays in 83% of patients and dose reductions in 39% of patients (21). No patients have discontinued treatment thus far due to ocular toxicity.

### Patients’ Perspective and Experience of Belantamab Mafodotin

In patient-reported experience analyses of 104 patients during and following treatment in DREAMM-2, 57% of patients reported some degree of visual problems whereas 40% reported symptoms of eye irritation (dry or itchy eyes and foreign body sensation) (22). About 12% of patients reported eye pain/soreness and burning eyes. Among 26 patients who were interviewed at the end of treatment, six patients considered stopping the treatment and two of those reported an actual discontinuation based on ocular symptoms. Despite ocular complaints, patients reported high satisfaction while on treatment and expressed desire to remain on treatment (22). In patient-reported outcome (PRO) measures of DREAMM-2 (n=92), two vision-related PRO questionnaires including NEI-VFQ-25 and Ocular Surface Disease Index (OSDI) were used to evaluate the patients’ symptoms and visual function both at baseline and every three weeks thereafter while on belamaf. 49.5% of patients reported 12.5-point or greater worsening on OSDI from baseline with median time to worsening of 44 days. Meaningful recovery of self-reported changes from the worst post-baseline severity was reported in 72% of patients with median time to improvement at 24 days (23).

### Mitigation Strategy for Ocular Toxicity

In previous clinical trials, the use of topical corticosteroids to mitigate MMAF-related ocular toxicity showed some benefit (10, 24). Borrowing the same concept, corticosteroid eye drops were used in DREAMM-1 for 4 days starting one day before the first dose of belamaf and then before subsequent doses. As there was no clear benefit from the use of steroids in DREAMM-1, DREAMM-2 ocular sub-study (n=30) further investigated the role of steroid eye drops. The duration of topical steroids was increased from 4 days to 7 days in DREAMM-2, but their use remained ineffective in preventing corneal changes (7). Apart from being an ineffective preventive option, topical steroids may potentially cause secondary ocular AEs. The long-term follow-up of five patients in DREAMM-1 showed the development of secondary cataracts and glaucoma due to frequent steroid use requiring cataract extractions and ocular pressure lowering drugs (25). We do not recommend use of steroid eye drops as a mitigation strategy to reduce ocular toxicity of belamaf. We also recommend baseline ophthalmic examination prior to the first dose and then before subsequent doses even in the absence of symptoms per DREAMM-2 protocol (11). Though baseline eye examination and then symptom-triggered evaluations were previously thought to be sufficient to monitor ocular toxicity, the increasing evidence of asymptomatic corneal toxicity warrants ophthalmic examination before each dose to detect early corneal changes (22). To complement ophthalmic examinations, NEI-VFQ-25 and OSDI questionnaires may be used to document quality of life changes but should not be used as the only guide to treatment modifications (26). Among 17 interviewees from DREAMM-1 who received 3.4 mg/kg of belamaf, about 76% had blurred vision but 93% of them did not consider it bad enough to discontinue the treatment (27). Therefore, prescribers should incorporate the objective evidence of ocular toxicity such as the *KVA* scale into treatment-related decisions. Using this scale, mild, moderate, severe superficial keratopathy and corneal epithelial defects on slit lamp correspond to G-1 to G-4 toxicity, respectively. Decline in BCVA from baseline up to 1 line, 2-3 lines, >3 lines and 20/200 as determined by Snellen chart correspond to G1-4 ocular toxicity (11). The role of cooling eye masks and vasoconstrictors prior to the belamaf infusion is unclear and should be used at the discretion of prescriber. Cooling eye masks were used in DREAMM-2 prior to each drug infusion to reduce corneal concentration. The philosophy behind such measures is to reduce the diffusion of belamaf into the cornea and therefore minimize the ocular concentration (25, 28). We strongly recommend the use of preservative-free artificial tears among all patients at least four times a day from the day of belamaf infusion and throughout the treatment course. Patients with pre-existing corneal diseases are at higher risk of ocular toxicity. Therefore, the use of belamaf in such population is cautioned and the use of contact lens should be avoided. Patients with baseline ocular abnormalities and prior corticosteroid or bortezomib use (98-100% of belamaf population) should be monitored closely with more extensive screening and regular ocular examination.

## Discussion

Belamaf-induced ocular toxicity is managed with dosage modifications (dose delays, reductions) or discontinuations in addition to preservative-free artificial tears (29). **Table 1** shows the incidence of keratopathy, and treatment changes in DREAMM-1, 2 and 6 trials.

**Table 1:** Summary of belantamab mafodotin related treatment changes in DREAMM (1, 2, 6) trials due to ocular toxicity.

Trial	Belamaf cohort/arm	Incidence of keratopathy	Time to onset of keratopathy	Time to resolution of keratopathy	Treatment holidays	Dose reduction	Discontinuation of therapy
			Median days (range)	Median days (range)			
DREAMM-1 (4, 5)	Dose-expansion cohort (3.4 mg/kg)	69% (n=24/35)	23 (1–84)	35 (5-442)	49% (n=17/35)	46% (n=16/35)	2.9% (n=1/35)
DREAMM-2 (6, 15)	Cohort1 (2.5 mg/kg)	70% (n=67/95)	36 (19–143)	71 (57–99)	47% (n=45/95)	23% (n=22/95)	1% (n=1/95)
	Cohort2 (3.4 mg/kg)	74% (n=74/99)	23 (9–150)	96 (70–127)	48% (n=48/99)	27% (n=27/99)	3% (n=3/99)
DREAMM-6 (18)	Arm B (belamaf 2.5 mg/kg + Bortezomib-dexamethasone)	100% (n=18/18)	NA	NA	83% (n=15/18)	39% (n=7/18)	0% (n=0/18)

NA, not available.

**Figure 1:**
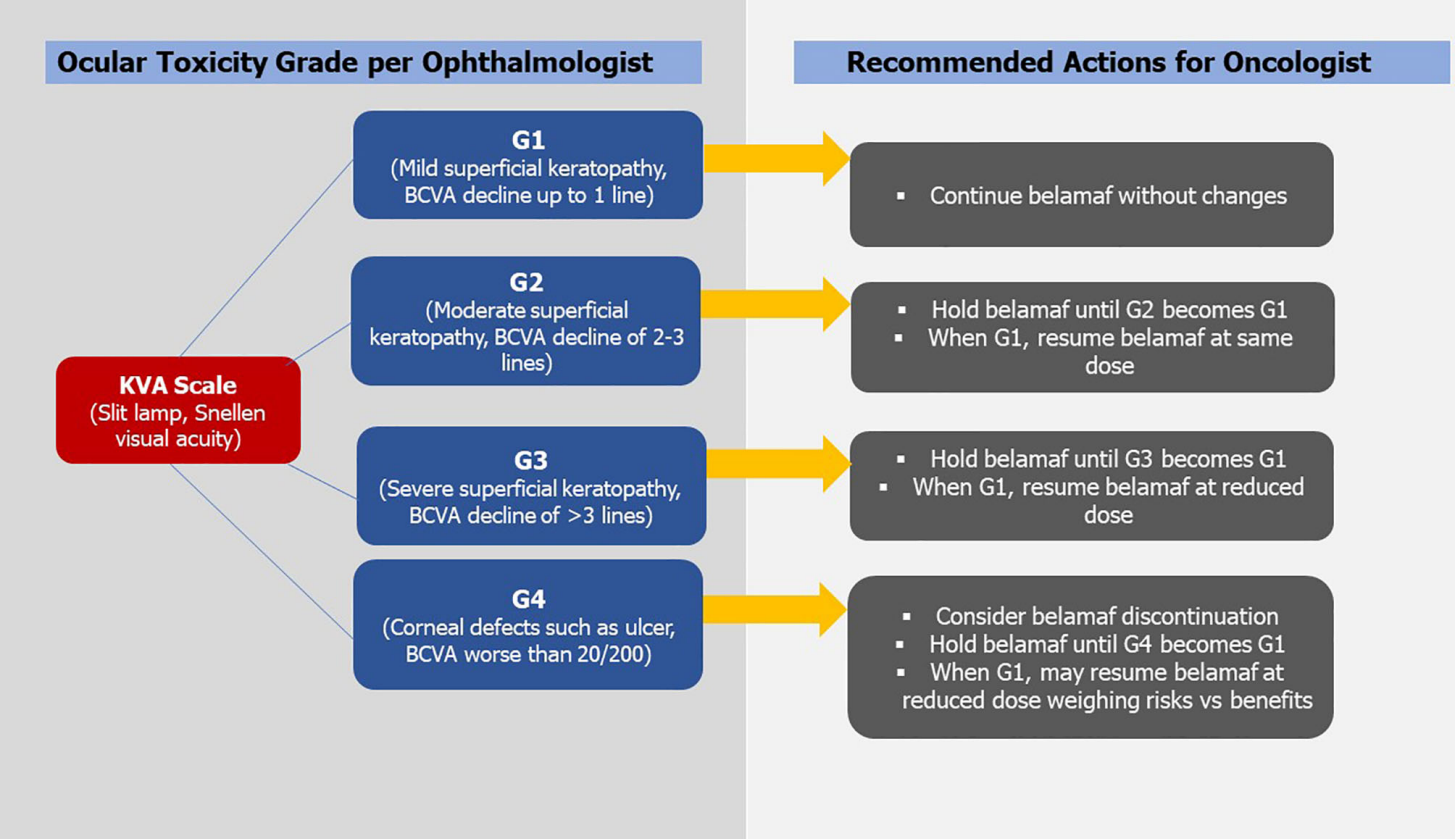
Showing Keratopathy and Visual Acuity Scale (*KVA*) and recommended treatment modifications of Belantamab Mafodotin (BCVA, best corrected visual acuity; belamaf, belantamab mafodotin; G, grade. *Figure is derived from DREAMM-2 guidelines).

Previously many studies have documented the resolution of corneal changes associated with MMAF use after treatment changes (9, 10).We recommend that belamaf-induced ocular toxicity be managed with close ophthalmology and hematology-oncology follow-ups and use of the *KVA* scale for treatment guidance. So far there are a few reports of permanent discontinuation of belamaf based on ocular toxicity, but no case of permanent blindness has been reported. In DREAMM-1, two patients discontinued belamaf in part-1 and one discontinued belamaf in part-2 due to ocular toxicity (5, 6). Among 66% of dose reductions in part-2, blurred vision was the cause in 34% of cases whereas it caused dose interruption or delays in 40% of cases (6). Keratitis and photophobia caused belamaf interruption or delays in 9% of cases for each category. Overall, corneal events in part-2 were responsible for dose reductions in 46% and dose interruptions/delays in 49% of participants (6). In DREAMM-2, keratopathy led to belamaf discontinuation in 1% and 3% of cases in 2.5 mg/kg and 3.4 mg/kg cohorts, respectively. Keratopathy caused dose reduction in 23% and 27% of cases in 2.5 mg/kg and 3.4 mg/kg cohorts whereas it caused dose delays or interruptions in 47% and 48% cases, respectively (7). Corneal findings identified on either KVA or BCVA assessments can be used to make treatment modifications (11). The worst *KVA* grade in the worst eye should guide such changes. For G-1 *KVA* toxicity, belamaf may be continued without any modification. For G-2/G-3 toxicity, belamaf should be stopped until corneal/BCVA findings return to ≤ G-1. For G-2, belamaf can be resumed at the same dose but for G-3, belamaf should be resumed at a reduced dose ensuring that G-2 or G-3 ocular toxicity has improved to ≤ G-1. The G-4 *KVA* findings may require belamaf discontinuations. If the decision based on risk *vs* benefit assessment is to resume belamaf, it should be resumed at a reduced dose after the G-4 toxicity has improved to ≤ G-1 (11). When reduction in belamaf dose is required, few authors have recommended a 25% reduction in the dose, i.e., from a standard approved dose of 2.5 mg/kg to ~1.9 mg/kg (7, 25). For judicious and transparent use of belamaf considering its ocular toxicity, GlaxoSmithKline devised a Risk Evaluation and Mitigation Strategy *(REMS)* program called *BLENREP REMS* that is endorsed by the FDA (1). *REMS* ensures that prescribers are certified in the program and wholesale distributors are distributing belamaf to certified entities only. Schematic guidance of treatment modification based on the *KVA* scale derived from DREAMM-2 and *BLENREP REMS* is shown in **Figure 1**.

## Conclusion

Belantamab mafodotin is associated with significant ocular toxicity especially in the presence of baseline ocular abnormalities. Serial ophthalmic examinations employing the *KVA* scale and artificial tears are the best strategies to mitigate toxicity. Ocular side-effects of belamaf should be managed with dose modifications, interruptions, or discontinuations. Patients should be monitored closely with ophthalmology and hematology-oncology follow-ups to ensure the safe use of belamaf.

## Author Contributions

AW developed the idea of study and formulated a study plan and manuscript writing. He was also involved in manuscript editing from the beginning till the end and his main section of the article were Mitigation Strategy for Ocular Toxicity and Discussion. AR helped in research strategy and literature review. KM and AM reviewed the literature and wrote the introduction section. HE, MK, and AK were involved in writing the subsections Mechanism of Ocular Toxicity, Presentation of Ocular Toxicity, and Patients’ Perspective and Experience of Belantamab Mafodotin, respectively. All authors contributed to the article and approved the submitted version.

## Conflict of Interest

The authors declare that the research was conducted in the absence of any commercial or financial relationships that could be construed as a potential conflict of interest.
